# Relationship between allergic diseases and mental disorders in women: A systematic review and meta-analysis

**DOI:** 10.3389/fpsyt.2022.1026032

**Published:** 2022-11-09

**Authors:** Lisha Liu, Chao Luo, Mengni Zhang, Xudong Ao, Huixia Liu, Shunlin Peng

**Affiliations:** ^1^School of Clinical Medicine, Chengdu University of Traditional Chinese Medicine, Chengdu, China; ^2^Department of Otolaryngology, Hospital of Chengdu University of Traditional Chinese Medicine, Chengdu, China

**Keywords:** allergic diseases, mental disorders, women, relationship, meta-analysis

## Abstract

**Background:**

The relationship between allergic diseases (AD) and mental disorders (MD) in women has not been fully systematically evaluated. We aimed at validating this correlation.

**Methods:**

The relevant cohort and case-control studies from the establishment of the database to February 18, 2022 in PubMed, Embase, and Cochrane library were searched by computer. The researchers conducted the quality evaluation of the included articles by reviewing and discussing with reference to relevant standards, and conducted the analysis of the correlation between female patients with AD and MD by using Review Manager 5.4.

**Results:**

Six observational studies from 2631 studies (n = 1160858 women) were assessed as medium and high-quality studies. The meta-analysis demonstrated that AD was correlated with MD in female patients (OR = 1.21, 95%CI: 1.14–1.29), including asthma (OR = 1.16, 95%CI: 1.11–1.22), allergic rhinitis (OR = 1.31, 95%CI: 1.06–1.63), and atopic dermatitis in women (OR = 1.37, 95%CI: 1.24–1.50) were associated with MD. At the same time, subgroup analysis was performed according to region, study design, criteria of AD and MD, and the results demonstrated that both AD and MD were correlated in these different conditions.

**Conclusion:**

Allergic diseases in female patients do have an association with mental disorders.

**Systematic review registration:**

[https://www.crd.york.ac.uk/PROSPERO/], identifier [CRD42022311146].

## Introduction

Allergic diseases (AD) are a series of health issues that are widely concerned worldwide. The incidence rate of AD is increasing year by year. Heredity and environment are the main pathogenic factors, while pathogenesis is mainly “T helper type 2 immune response” (1). In addition, “atopic march” is more common in AD (2). Among these, the global incidence rate of allergic rhinitis (AR) is 10–40% (3), atopic dermatitis and asthma also affect 230 million and 300 million people worldwide (4, 5). According to existing research, allergies gradually become serious with the growing of girls, so lifelong allergic diseases are more obvious in women (6).

Mental disorders (MD) are a global problem, which have a crucial impact on human health. They usually include depression, anxiety, etc. (7). The prevalence of depression and anxiety in women is 1.5–2.5 times higher than that in men (8). Additionally, gender-specific disorders are more significant in women, such as perinatal MD (9). It is reported that the disease mechanism of inflammatory dysregulation may lead to MD (10, 11). Thus, the possible association between AD and MD has been widely concerned by society.

The association between AD and MD has been controversial in recent years. One study showed a correlation between AR and depression (5). Another studies illustrated that patients with atopic dermatitis had a higher incidence of depression than those with non-atopic dermatitis (12). In addition, the study found that women with depression or anxiety had a higher incidence of uncontrolled asthma during pregnancy than women without depression or anxiety. The incidence of uncontrolled asthma during at least one or more than two study visits was 52.9% vs. 32.7% and 23.5% vs. 9.6%, respectively (13). It was reported that major depressive disorder had more adverse effects on the severity of asthma and AR than non-major depressive disorder (14). Nevertheless, a systematic evaluation found that asthma did not have an association with depression (15). Another prospective study found no association between AD and depression (16). In conclusion, the existing systematic evaluation mainly targets a class of diseases in AD and MD, and there is a lack of studies specifically on women. At present, the relationship between female AD and MD has not been clarified. Therefore, this study intends to examine the association by analyzing existing observational studies.

## Methods

The preferred reporting items for systematic review and meta-analysis (PRISMA) were used in this study (17). We registered on the PROSPERO (NO. CRD42022311146).

### Search strategy

English articles published in PubMed, Embase and Cochrane Library from inception to February 18, 2022, were searched. The combination of subject words and free words is adopted. Search terms include mental disorder terms (“mental disorder,” “psychiatric disorder,” “psychiatric diagnosis,” etc.) and terms of allergy and atopic diseases (“allergic rhinitis,” “asthma,” “hay fever,” “Eczema,” “atopic dermatitis,” etc.) and female terms (“women,” “girls,” “women’s group,” etc.) and related terms (“risk,” “mortality,” “cohort,” etc.). Meanwhile, we also discuss the references in the main study with peer experts for additional reference information.

### Study selection and eligibility criteria

After eliminating the repeated and irrelevant articles, two researchers screened out the articles that met the standard by reading the title, the abstract, and the full text to finally determine the included articles. In case of disagreement and consensus cannot be resolved, a third researcher will assist in the review. Furthermore, if more information is required, we will contact the relevant authors to obtain it. We formulated the following inclusion criteria: (1) the type of study was an observational study (cohort study and case-control study); (2) the subjects were female patients with AD and MD; (3) comparison with the control group was involved; (4) it had scientific disease diagnostic criteria; (5) data such as odds ratio (OR) or relative rate (RR) and 95% confidence interval (CI) could be obtained. We excluded studies under the following circumstances: (1) articles published repeatedly or unable to obtain the full text; (2) data was incomplete or cannot be converted; (3) review, conference abstract, case report, and animal experiments; (4) study design, outcome indicators, and themes were inconsistent with the study; (5) there was no scientific diagnostic criteria for the disease.

### Data extraction and quality evaluation

A researcher extracted the information included in the study, including (1) basic information such as author, year, country, participant, and age; (2) study design and source of case samples; (3) diagnostic criteria for AD and MD. It was subsequently reviewed by another researcher, and differences were resolved through discussion, with the assistance of a third researcher. The quality of the included studies was evaluated by the Newcastle Ottawa scale (NOS) (18). There were three parts, including eight items, with a total score of 9 points. A score of ≥7 was rated as high quality, otherwise as low to medium quality. The evaluation was accomplished by two researchers reaching a consensus through discussion.

### Data synthesis

Review Manager 5.4 was used for meta-analysis of the included studies. Effect sizes of OR were pooled using generic inverse variance. Heterogeneity was indicated by I^2^ test. For low-degree heterogeneity (*P* < 0.10 and *I*^2^< 50%), the fixed-effects model was used. Otherwise, the random-effects model was selected (*P* < 0.10 and *I*^2^≥ 50%). Meanwhile, the source of heterogeneity was determined by subgroup and sensitivity analysis. If more than ten articles were included, the funnel plot was used to analyze the risk of bias.

## Results

### Study selection

**FIGURE 1:**
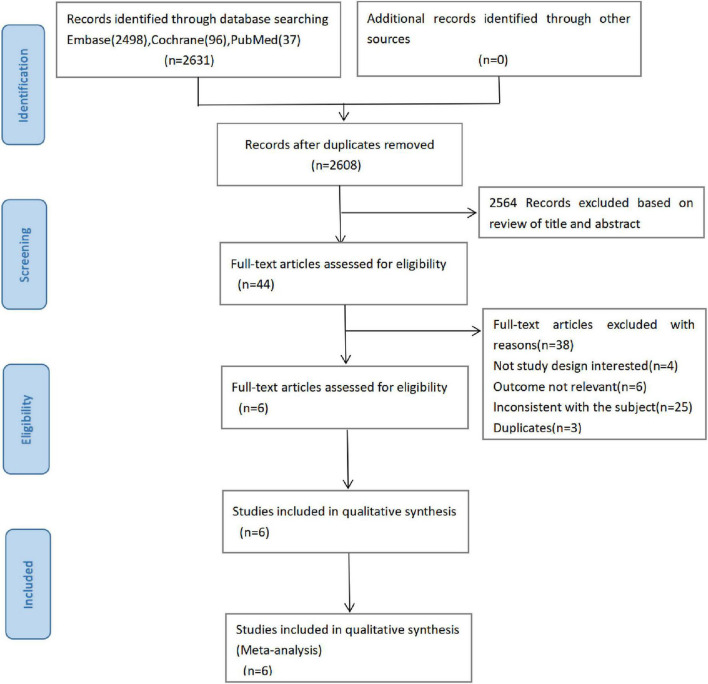
PRISMA flow chart of literature screening.

A total of 2631 articles (Embase 2498, Cochrane 96, PubMed 37) were initially detected through database retrieval, of which 23 duplicate articles were excluded. After reading the title and abstract, 2564 articles were excluded, and the remaining 44 articles were reviewed in full text. Finally, a total of 6 articles were included for analysis (Figure 1) (13, 19–23).

### Study characteristics

The 6 articles were published for 2014-2021 years, and all participants were women, with 189-846155 participants, respectively, from North America (Canada and USA) (19, 21, 23), Asia (Korea and Taiwan, China) (20, 22), and Oceania (Australia) (13). The age is 15-50 years old (19–22), but this information went unreported in 2 articles (13, 23). The included studies are all observational studies, five of which were cohort studies (13, 19, 21–23), and one was case-control study (20). Furthermore, the diagnostic criteria for AD include physician diagnosis (19–23) and questionnaire (13). The diagnostic criteria for MD include self-report (13), questionnaire (13, 22, 23) and physician diagnosis (19–21) (Table 1).

**TABLE 1:** Characteristics of 6 studies included in present study.

Author	Year	Country	Participants, N	Age(y)	Study design	Source of case samples	Criteria of allergic diseases	Criteria of mental disorders
Aker et al. (19)	2021	Canada	846155	15–49	cohort study	ICES	physician diagnosis	physician diagnosis
Yang et al. (20)	2021	Taiwan, China	199470	15–50	case-control study	NHIRD	physician diagnosis	physician diagnosis
Aker et al. (21)	2021	Canada	62583	15–49	cohort study	ICES	physician diagnosis	physician diagnosis
Kim et al. (22)	2020	Korea	20613	20–45	cohort study	KNHS	physician diagnosis	PHQ-9, JSQ
Grzeskowiak et al. (13)	2017	Australia	189	NA	cohort study	LMH	ACQ	self-report, ANRQ, EPDS
Coogan et al. (23)	2014	USA	31848	NA	cohort study	BWHS	physician diagnosis	CES-D

ACQ, Asthma Control Questionnaire; ANRQ, Antenatal Risk Questionnaire; BWHS, Black Women’s Health Study; CES-D, Center for Epidemiological Studies–Depression Scale; EPDS, Edinburgh Postnatal Depression Score Questionnaire; ICES, Institute for Clinical Evaluative Sciences; JSQ, Jenkins Sleep Questionnaire; KNHS, Korean Nurses’ Health Study; LMH, Lyell McEwin Hospital; NHIRD, National Health Insurance Research Database; PHQ-9, Patient Health Questionnaire.

### Quality assessment

The quality of included studies was evaluated according to NOS. There were 5 articles of high quality, and the other one (22) was rated as medium quality, due to the exposed group was not representative and merely Korean feminine nurses were taken as the research object (Tables 2, 3).

**TABLE 2:** Results of quality evaluation using the NOS for included cohort studies.

Study	Selection				Comparability	Outcome			Quality score
	Exposed groups	Unexposed groups	Ascertainment of exposure	Without outcome	Comparability	Evaluation	Enough time	Adequacy	
Aker et al. (19)	★	★	★	★	★★	★	✩	✩	7
Aker et al. (21)	★	★	★	★	★✩	★	✩	★	7
Kim et al. (22)	✩	★	★	★	★✩	✩	★	★	6
Grzeskowiak et al. (13)	★	★	★	★	★★	★	★	★	9
Coogan et al. (23)	★	★	★	★	★✩	★	★	★	8

★: This option has a score of 1; ✩: This option has a score of 0.

**TABLE 3:** Results of quality evaluation using the NOS for included case-control study.

Study	Selection				Comparability	Outcome			Quality score
	Adequate definition	Representa -tiveness	Selectivity of controls	Determination of controls	Comparability	Exposure factors	Same method	Non-response rate	
Yang (20)	★	★	★	★	★★	✩	★	★	8

★: This option has a score of 1; ✩: This option has a score of 0.

### The association between allergic diseases and mental disorders

**FIGURE 2:**
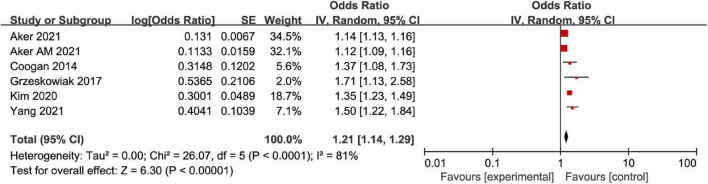
Forest map of association meta-analysis between AD and MD.

**FIGURE 3:**
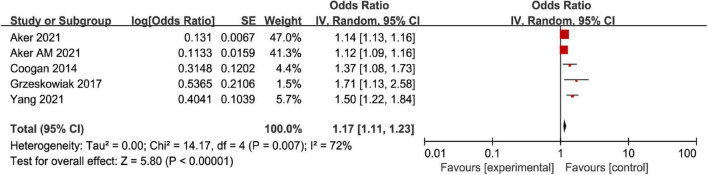
Forest map of association meta-analysis between AD and MD—Only relatively high-quality studies were included.

**FIGURE 4:**
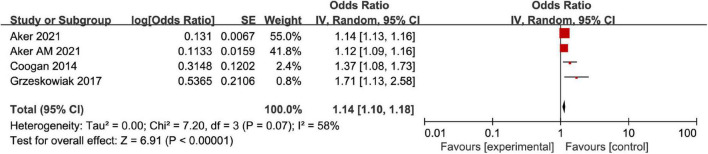
Forest map of association meta-analysis between AD and MD—Only studies of the same disease type were included.

The correlation between AD and MD was analyzed for the 6 included articles (13, 19–23), and significant heterogeneity was found through test (*I*^2^ = 81%, *P* < 0.001). The analysis of random-effects model showed that AD in female patients was correlated with MD (OR = 1.21, 95%CI: 1.14–1.29, *P* < 0.001) (Figure 2). Considering that the causes of significant heterogeneity were related to different types of included diseases and low quality of literature. Therefore, a moderate-quality study of Kim (22) was excluded by the sensitivity analysis, and the results showed that OR = 1.17, 95%CI: 1.11–1.23, *P* < 0.001, and heterogeneity decreased (*I*^2^ = 72%, *P* = 0.007) (Figure 3). Excluding the studies of Yang and Kim (20, 22) with inconsistent disease types, the sensitivity analysis showed that OR = 1.14, 95%CI: 1.10–1.18, *P* < 0.001, and heterogeneity significantly decreased (*I*^2^ = 58%, *P* = 0.07) (Figure 4).

### Subgroup analysis

**FIGURE 5:**
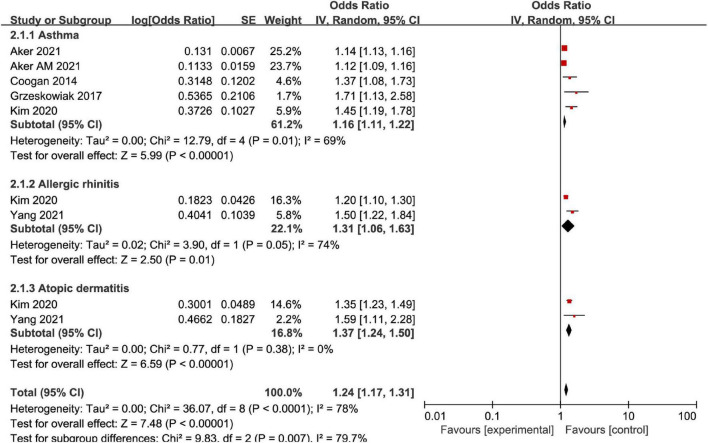
Forest map of the relationship between AD disease types and MD.

For different disease types of AD, the subgroup analysis illustrated heterogeneity in the asthma group (*I*^2^ = 69%, *P* = 0.01), and asthma was associated with MD (OR = 1.16, 95%CI: 1.11–1.22, *P* < 0.001). There was heterogeneity in AR group (*I*^2^ = 74%, *P* = 0.05), and the incidence of mental disorders in AR group was higher than that in non-AR control group (OR = 1.31, 95%CI: 1.06–1.63, *P* = 0.01). There was no significant heterogeneity in the atopic dermatitis group (*I*^2^ = 0%, *P* = 0.38), and atopic dermatitis was associated with MD (OR = 1.37, 95%CI: 1.24–1.50, *P* < 0.001). Therefore, female patients with AD were correlated with MD (Figure 5).

Meanwhile, we conducted subgroup analysis of region, study design, criteria of AD and MD, and the results demonstrated that under the different conditions mentioned above, AD and MD were correlated. Furthermore, in the region and criteria of MD subgroup analysis, we found that Asian region (OR = 1.38, 95%CI: 1.26–1.50, *P* < 0.00; *I*^2^ = 0%) and questionnaire for diagnosis of MD (OR = 1.37, 95%CI: 1.25–1.49, *P* < 0.001; *I*^2^ = 0%) without significant heterogeneity (Table 4).

**TABLE 4:** Meta-analytical results and results of the subgroup analyses on the association between allergic diseases and mental disorders.

	N studies	Subgroup analysis	
		OR[95%CI] (*P*-value)	Heterogeneity-I^2^;*P*-value
**Region**			
North America	3	1.14 [1.12, 1.15] (<0.001)	42%;0.18
Asia	2	1.38 [1.26, 1.50] (<0.001)	0%;0.37
Oceania	1	1.71 [1.13, 2.58] (=0.01)	–
**Study design**			
Cohort study	5	1.19 [1.12, 1.26] (<0.001)	79%;0.0007
Case-control study	1	1.50 [1.22, 1.84] (<0.001)	–
**Criteria of allergic diseases**			
Physician diagnosis	5	1.20 [1.13, 1.27] (<0.001)	82%;0.0002
Questionnaire	1	1.71 [1.13, 2.58] (=0.01)	–
**Criteria of mental disorders**			
Physician diagnosis	3	1.14 [1.10, 1.20] (<0.001)	75%;0.02
Questionnaire	3	1.37 [1.25, 1.49] (<0.001)	0%;0.55

“–”: No heterogeneity computed, as there is one single study in this subgroup.

## Discussion

A previous meta-analysis found that adolescents with atopic dermatitis took a higher risk of MD in comparison with non-atopic dermatitis (OR = 1.652; 95% CI: 1.463–1.864) (24). Another systematic evaluation found that AR was associated with MD, and the risk of anxiety and depression in AR patients was higher than that in the non-AR group (25). Moreover, a systematic evaluation of the association between asthma and anxiety was conducted in 2021 based on observational studies, which found the association between asthma and anxiety disorders (OR = 2.08; 95% CI: 1.70–2.56) (26). Nevertheless, the above meta-analysis was limited to one or two types of diseases and failed to conduct a comprehensive review of a series of disease problems and gender factors. At present, there is still a lack of comprehensive evaluation of the relationship between AD and MD in women. Therefore, based on six observational studies, the systematic evaluation in this work found a correlation between AD and MD in women. In addition, subgroup analysis also illustrated that women with AD did have an association with MD. Among them, asthma, atopic dermatitis and AR were mainly associated with depression (13, 19, 20, 22, 23). Secondly, AR and atopic dermatitis would also be combined with sleep disorders (22). Women with asthma were also accompanied by other MD, such as anxiety disorders (13, 19). In addition, depression/anxiety in MD had the strongest correlation with uncontrolled asthma during pregnancy (RR = 1.71, 95%CI: 1.13–2.58) (13).

At present, the pathogenesis of AD is still being explored. Some theories suggest that inflammatory cytokines (tumor necrosis factor-α, interleukin, etc.) in AD bound to receptors on nerve cells, which can reduce the volume of gray matter in the hippocampus and thus have an impact on depression (27). Besides, the hypothalamic-pituitary-adrenal (HPA) axis activated in the course of inflammation is also associated with MD such as depression (28). Moreover, α-amylase and salivary cortisol, as well as a common NR3C1 gene, are found to correlate asthma with anxiety (29, 30). Of course, the sex-specific disease is also more and more apparent in women, particularly perinatal women. With the change in hormone level and immune function, the susceptibility to MD is gradually obvious (31). Therefore, society pays special attention to the relationship between female AD patients and MD. Clinically, we should first understand whether women with AD have a medical history and family history related to MD, so as to carry out comprehensive treatment. Secondly, especially for perinatal women, AD should be monitored and intervened in time to avoid the emergence of postpartum MD. Furthermore, treatment modalities that may cause side effects on the mental system should be avoided when treating AD. Finally, in addition to the use of medication, effective psychological intervention measures should be taken to prevent the emergence of self-harm or suicide.

## Strengths and limitations

This systematic evaluation is mainly aimed at the female group at first. The perinatal women and professional women are comprehensive evaluated, so as to explore the impact of disease on women. Secondly, the diseases studied are not limited to a single type, but a larger range of diseases are discussed for AD and MD, and a series of diseases such as AR, anxiety, and depression are comprehensively analyzed. Thirdly, all the included studies were rated as medium and high quality after NOS quality evaluation. Fourthly, compared with the 106813 samples studied by Ye et al. (26), we analyzed more than 1.16 million participants, involving Asia, North America, Oceania and other regions around the world, which provided a reliable and sufficient basis for this meta-analysis.

This study also has the following limitations. First, the observational studies principally focused on cohort studies and involved merely one case-control study. Second, AD was predominant in asthma. The relevant AR and atopic dermatitis solely involved two studies respectively, and the severity of the disease was not analyzed. Third, the OR, RR and 95%CI obtained from the paper failed to completely exclude relevant confounding factors. Fourth, there may be some bias in reviewing only those published in English and excluding other forms of reporting. Fifth, three of the six articles used questionnaire assessment in the criteria of MD.

## Conclusion and future directions

In conclusion, our meta-analysis finds a significant association between AD (AR, asthma, and atopic dermatitis) and MD in women. Future studies require adjusting for confounding variables and include a larger number of high-quality studies. Additionally, multi-center randomized controlled trials are needed to further explore pathogenesis and clinical prevention and treatment of the disease.

## Data availability statement

The original contributions presented in this study are included in the article/supplementary material, further inquiries can be directed to the corresponding author.

## Author contributions

LL and SP participated in the design of this study. MZ, XA, and HL discussed and screened the literature. LL was responsible for data analysis and writing. CL and SP were responsible for revising and reviewing the manuscript. All authors contributed to the article and approved the submitted version.
